# Primary resistance to clarithromycin, metronidazole and amoxicillin of *Helicobacter pylori *isolated from Tunisian patients with peptic ulcers and gastritis: a prospective multicentre study

**DOI:** 10.1186/1476-0711-9-22

**Published:** 2010-08-13

**Authors:** Khansa Ben Mansour, Christophe Burucoa, Meriem Zribi, Afef Masmoudi, Sami Karoui, Lamia Kallel, Soufiène Chouaib, Samira Matri, Monia Fekih, Sonia Zarrouk, Mounir Labbene, Jalel Boubaker, Imed Cheikh, Mongi Ben Hriz, Nadia Siala, Abdelkarim Ayadi, Azza Filali, Nabil Ben Mami, Taoufik Najjar, Ahmed Maherzi, Mohamed Tahar Sfar, Chedlia Fendri

**Affiliations:** 1 Microbiology laboratory/UR04SP08 Rabta University Hospital-Tunis, 1007 El Jabbari, Tunisia; 2 EA 4331 « Laboratoire Inflammation Tissus Epithéliaux Cytokines », 86021 Poitiers, France; 3 Gastroenterology A unit, Rabta University Hospital-Tunis, 1007 El Jabbari, Tunisia; 4 Gastroenterology B unit, Rabta University Hospital-Tunis, 1007 El Jabbari, Tunisia; 5 Gastroenterology unit, Regional hospital of Menzel Bourguiba, 7050, Tunisia; 6 Pediatric unit Mongi Slim University Hospital, La Marsa, 2070 Tunis, Tunisia; 7 Pediatric unit, Mahdia Regional Hospital, 5100, Tunisia; 8 Gastroenterology unit, Charles Nicolle University Hospital, Bd 9Avril, 1006 Bab Souika, Tunis, Tunisia

## Abstract

**Background:**

The frequency of primary resistance to antibiotics in H. pylori isolates is increasing worldwide. In Tunisia, there are limited data regarding the pattern of H. pylori antibiotic primary resistance.

**Aim:**

To evaluate the primary resistance of H. pylori to clarithromycin, metronidazole and amoxicillin and to detect the mutations involved in clarithromycin resistance.

**Materials and methods:**

273 strains isolated from adults and children were enrolled. The primary resistance to clarithromycin, metronidazole and amoxicillin was evaluated by means of E-test minimal inhibitory concentration (MIC). The real-time PCR using Scorpion primers was performed in all cases to assess clarithromycin primary resistance and point mutations involved.

**Results:**

No resistance to amoxicillin was detected. For adults, resistance to clarithromycin and metronidazole was found respectively in 14.6% and 56.8%, and respectively in 18.8% and 25% in children. Overall, the rates of global primary resistance to clarithromycin and metronidazole in Tunisia were respectively determined in 15.4% and 51.3%.

By the use of Scorpion PCR, the A2143G was the most frequent point mutation observed (88.1%), followed by the A2142G (11.9%); the A2142C was not found and 18 of 42 patients (42.8%) were infected by both the resistant and the susceptible genotype.

The association of clarithromycin resistance with gender was not statistically significant, but metronidazole resistant strains were isolated more frequently in females (67.8%) than in males (32.2%) and the difference was significant. As for gastroduodenal diseases, the difference between strains isolated from patients with peptic ulceration and those with non peptic ulceration was not statistically significant. When about the distribution of resistant strains to clarithromycin and metronidazole between the three Tunisian cities (Tunis, Menzel Bourguiba and Mahdia), the difference was not statistically significant.

**Conclusion:**

Local data regarding the primary resistance of H. pylori to clarithromycin, metronidazole and amoxicillin and the main genetic mutation involved in clarithromycin resistance in vivo (A2143G) are necessary to prove a clear need for a periodic evaluation of antibiotic consumption and new therapeutic strategies in Tunisia in order to avoid the emergence of resistant strains.

## Introduction

*Helicobacter pylori *(*H. pylori*) colonizes the human stomach and it has emerged as an important pathogen in the field of gastroenterology [1]. In Tunisia, his prevalence is average 90% in peptic ulcer [2]. Since 2005, the Tunisian consensus had recommended the eradication of *H. pylori *by a triple therapy which includes amoxicillin, clarithromycin or metronidazole combined with proton pump inhibitors (PPI) for 7 to 10 days [3]. Resistance to these drugs reduces the success rate of treatment regimens both in adults and children. Several studies have demonstrated that primary resistance to clarithromycin is a major predictive factor for therapeutic failure [4]; the mechanism of this resistance decreased binding of the antibiotic to the 50 S ribosomal subunit of the microorganism and is due to three distinct point mutations (A2142G, A2143G and A2142C) within the peptidyltransferase region encoded in domain V of the *H. pylori *bacterial 23 S rRNA gene [5]. The detection of mutations was performed by the use of several real-time PCR methods [5-9]. Resistance to metronidazole is mainly due to mutations in the *rdxA *gene encoding RdxA, an oxygen-insensitive nitroreductase [10]. When about primary resistance to metronidazole, conflicting results have been reported on its impact in the treatment outcome [11]. Prevalence rates of primary clarithromycine and metronidazole resistance were documented to be higher in developing countries than in industrialized ones [12,13]. Because of limited data on the resistance of *H. pylori *to antibiotics in Tunisia, the aims of the present prospective and multicentre study were: (i) to evaluate, by means of E-test and Scorpion PCR the prevalence of primary resistance to clarithromycin, and by means of E-test, the rates of primary resistance to metronidazole and amoxicillin of 273 clinical strains isolated from children and adults, (ii) to detect, for the first time in Tunisia, the mutations involved in clarithromycin resistance by Scorpion PCR as previously described [14].

## Materials and methods

### Materials

#### Patients and biopsies

In this study, the biopsy samples were taken over a 2 years-period (March 2005 to August 2007) from patients referred for endoscopy at 6 different units of gastroenterology in three Tunisian cities (Tunis, Menzel Bourguiba and Mahdia). 273 patients had *H. pylori *positive culture; 48 children (aged from 2 to 14 years; mean age 8.75 years) were distributed into gastritis in 47 cases and one case of duodenal ulcer and, 225 adults (aged from 18 to 88 years; mean age 38.3 years) in which 148 cases were defined as gastritis, 66 cases as duodenal ulcer and gastric ulcer was observed in 11 cases. All patients had not been treated before. Regarding the total group, patients were distributed into gastritis in 195 cases, duodenal ulcer in 67 cases and gastric ulcer in 11 cases. The biopsy specimens were cultured on Columbia agar plates supplemented with 10% horse blood and Skirrow supplement (Oxoid, England) under microaerobic conditions for a maximum of 6 days as described by Ben Mansour et al [15].

#### Setting

6 gastroenterology centers in 3 Tunisian cities: 1/Tunis ( Adult Units A and B in Rabta University Hospital, Adult Gastroenterology Unit-Charles-Nicolle University Hospital, and Paediatric Unit in Mongi-Slim University Hospital); 2/Adult Gastroenterology Unit-Menzel Bourguiba Regional Hospital, and 3/Paediatric Unit-Mahdia Regional Hospital, between March 2005 and August 2007.

#### DNA extraction

Genomic DNA was extracted with the QIAamp DNA mini kit (Qiagen, Germany) according to the manufacturer's instructions. The isolated DNA was eluted in 200 μl of 1 × TE buffer (10 mM Tris-HCl, 1 mM EDTA [pH 8.0]) and stored at -20°C until use.

#### MIC determination

The inoculum was adjusted to an opacity equivalent to a 3-4 McFarland turbidity standard and was flooded on Columbia agar plates containing 10% horse blood. The E-test strip (AB Biodisk, Sweden) was placed on the plate when it was dry and according to the instructions of the manufacturer. Incubation was performed in a microaerobic atmosphere for 48 to 72 hours at 37°C. Strains were considered resistant to clarithromycin when the MIC was ≥ 1 μg/ml for clarithromycin, ≥ 8 μg/ml for metronidazole and ≥ 0.5 μg/ml for amoxicillin. These breakpoints were used on the basis of recommendations as previously described [16].

#### Determination of point mutations in the 23 S rRNA gene of *H. pylori *by Scorpion PCR

A 140-bp fragment of the 23 S rRNA gene of 273 strains of *H. pylori *was amplified by using primer 23SF2 and scorpion primers (Table 1). By using the Smart Cycler 2.0 thermocycler, the PCR and hybridization reactions were carried out in a volume of 25 μl with the Premix Ex Taq (TAKARA, Shiga, Japan), 1 μl of extracted DNA from culture (approximately 200 ng), 0,1 μM of primer 23SF2, 0,14 μM of 23Ssc A2142G, 0,18 μM of 23Ssc A2143G, 0,1 μM of 23Ssc A2142C and 0,08 μM of 23Ssc WT. Amplification was performed after a denaturation step (95°C for 15s) for 50 cycles, annealing (55°C for 30s) and extension (72°C for 20s). Four channels were reading the fluorescence for each sample: 6-carboxyfluorescein [FAM], Texas red, Cy3 and Cy5. Data analysis was performed with Cepheid Software (Cepheid, Sunny vale, CA). Four strains of *H. pylori *were used as positive controls: one reference strain (J99) with the wild type genotype/phenotype and three Clarithromycin-resistant strains (HP 825, HP 225 and HP 222) with mutations determined by sequencing of the 23 S rRNA gene (mutations A2142C, A2142G and A2143G, respectively) [14].

**Table 1:** Scorpion primers and primer sequences

Oligonucleotide	Mutations	Sequence
23SF2	-	5'-TGCGAAATTCCTTGTCGG-3'
23SSc	WT	5'-FAM AAGGTAGGTGAAAATTCCTCC TACC BHQ1 HEG GGACCACGGGGTCTTT-3'
23Ssc	A2143G	5'-Texas red AAGGTAGGTGAAAATTCCTCC TACC BHQ2 HEG GGACCACGGGGTCTTT-3'
23Ssc	A2142G	5'-Cy3 AAGGTAGGTGAAAATTCCTCC TACC BHQ1 HEG GGACCACGGGGTCTTC-3'
23Ssc	A2142C	5'-Cy5 AAGGTAGGTGAAAATTCCTCC TACC BHQ1 HEG GGACCACGGGGTCTTG-3'

### Statistical analysis

Data were analyzed using *X*^2 ^test. A *p *value of < 0.05 was considered to be statistically significant.

## Results

### Detection of resistance to clarithromycin, metronidazole and amoxicillin by E-test

In adults, the rates of primary resistance to clarithromycin and metronidazole were determined respectively in 14.6% (33/225) and 56.8% (128/225) (Table 2). Primary resistance to both clarithromycin and metronidazole was found in 12.9% (29/225) of cases.

**Table 2:** Distribution of resistant strains to clarithromycin, metronidazole and amoxicillin among children, adults and a total group

	Clarithromycin Resistance	Clarithromycin Susceptible	Metronidazole Resistance	Metronidazole Susceptible	Amoxicilline Resistance
Children (N = 48)	9 (18.8%)	39 (81.2%)	12* (25%)	36 (75%)	0
Adults (N = 225)	33 (14.6%)	192 (85.4%)	128* (56.8%)	97 (43.2%)	0
Total group (N = 273)	42 (15.4%)	231 (84.6%)	140 (51.3%)	133 (48.7%)	0

*: a significant difference is present

Among children, the primary resistance to clarithromycin and to metronidazole was found respectively in 9 cases (18.8%) and in 12 cases (25%) of all 48 isolates (Table 2). 8 strains (16.7%) were resistant to both clarithromycin and metronidazole.

Overall, the rates of global primary resistance to clarithromycin and metronidazole in Tunisia were respectively determined in 15.4% (42 of 273) and in 51.3% (140/273) and the rate of primary resistance to both clarithromycin and metronidazole resistance was 13.6% (37/273) in all studied strains. No resistance to amoxicillin was found in the present study.

### Detection of clarithromycin resistance and point mutations conferring resistance to clarithromycin by Scorpion PCR

In the present study, the primary resistance to clarithromycin was also determined by means of Scorpion PCR as reported elsewhere [14]. Resistance rate was 18.8% (9/48) among children and 14.6% (33/225) in adults. We have not found discrepancies between Scorpion PCR and E-test methods. Mutations detected in only 42 clarithromycin-resistant strains were summarized in Table 3. In 37 out of the 42 studied strains, A2143G was detected (88.1%), with 21.4% (8/37) in children and 78.6% (29/37) in adults. The A2142G was found in 11.9% (5/42) of strains; 2.3% was detected in children and 9.6% in adults. The prevalence of the two point mutations (A2143G and A2142G) did not statistically differ between adults and children. The A2142C mutation was not found in the present study and was also less common. By using Scorpion PCR, we revealed a presence of a mixed population in the same sample. Interestingly, 18 of 42 subjects (42.8%) were infected by both the susceptible and the resistant genotype.

**Table 3:** Relationship between mutations conferring clarithromycin resistance and pathologies

	NPU*	PU**	Total
A2143G	28 (75.6%)	9 (24.4%)	37
A2142G	4 (80%)	1 (20%)	5
A2142C	0	0	0

NPU*: non peptic ulceration; PU**: peptic ulceration

The distribution of primary resistance to clarithromycin and metronidazole according to gender and gastroduodenal diseases was shown in tables 4 and 5. As for association of clarithromycin resistance with gender, no significant difference in resistance was found (p > 0.05). In contrast to clarithromycin, metronidazole resistant strains were isolated more frequently in females than in males (67.8% vs. 32.2%) and the difference was statistically significant (p < 0.05).

**Table 4:** Relationship between the sex of patients and resistance rates

	Female	Male	Total
Clarithromycin resistance	26 (61.9%)	16 (38.1%)	42
Metronidazole resistance	95 (67.8%)*	45 (32.2%)*	140

*: a significant difference is present

**Table 5:** Relationship between the gastroduodenal diseases and resistance rates

	NPU	PU	Total
Clarithromycin resistance	32 (76.2%)**	10 (23.8%)**	42
Metronidazole resistance	96 (68.6%)**	44 (31.4%)**	140

**: the difference was not statistically significant

When about gastroduodenal diseases, no statistically difference was found between strains isolated from patients having peptic ulceration and those with non peptic ulceration (p > 0.05).

As for the distribution of resistant strains to clarithromycin and metronidazole among the 3 Tunisian cities (Table 6), the difference was not statistically significant.

**Table 6:** Resistance rates among the 3 Tunisian cities

	Tunis (N = 181)	Menzel Bourguiba (N = 83)	Mahdia (N = 9)	Total (N = 273)
Clarithromycin resistance	27**	12**	3	42
Metronidazole resistance	95**	42**	3	140

**: the difference was not statistically significant

## Discussion

In Tunisia, it's very important to know the rates of primary resistance of *H. pylori *strains to clarithromycin, metronidazole and amoxicillin, especially when antibiotic resistance in *H. pylori *represents a serious public health problem.

In adults, the rates of primary resistance to clarithromycin and metronidazole were documented as 17.5% for clarithromycin and 56% for metronidazole in 2002 [16]. There is a decrease in the rate of clarithromycin resistance with 14.6% founded in our present study and a slightly variation (56.8%) among metronidazole. In terms of clarithromycin primary resistance, our prevalence would seem higher than that computed in Malaysia (2.1%) [17], in Germany (2.2%) [18], in Canada (less than 4%) [19], in Hong-Kong (4.5%) [20], in Korea (5-6%) [21] and in one European multicentre study (9.9%) [11]. This prevalence was almost similar to those found in the USA (10-15%) [22], in France [23] but was lower to that detected in Iran (17%) [24], in Turkish (27.6%) [25] and in one European study (43.5%) [26]. This difference in clarithromycin resistance rate between several countries might be due to the prescription and administration of this antibiotic. Indeed, since clarithromycin is a widely used antimicrobial drug to treat infection in other organ systems than gastric tract, e.g., respiratory tract infections, the prevalence of clarithromycin-resistant *H. pylori *is increasing continuously. No differences in gastroduodenal diseases were seen between patients with non peptic ulceration and those with peptic ulceration (p > 0.05); the majority of studies have mentioned no differences in prevalence in accordance with disease status, but two studies did mention such differences, and they concluded that duodenal ulcer and non-ulcer dyspepsia patients should be managed differently in medical practice and considered independently in eradication trials [27]. A2143G and A2142G are the most prevalent point mutations, and studies have claimed that these mutations play a major role in clarithromycin resistance [5,23]. The prevalence distribution of A2143G (78.6%) and A2142G (9.6%) in adults was similar to those of patients in Taiwan study and in a prospective multicentre survey carried out in Europe [26]. We have not found the A2142C mutation which is less common, e.g., it was found in 5 of 129 resistant strains [5].

As for metronidazole resistance, it is well known that the prevalence of *H. pylori *is much higher in developing countries (50-80%). Our prevalence was much higher than that documented in an European multicentre study and in Bulgaria where the rates were evaluated respectively to 33.1% and 26.5% [11,28], but was lower to that computed in Columbia (82%) [29], in Mexico (76.3%) [30] and in Sweden (76%) [31]. In one Indian study, the resistance to metronidazole was evaluated to 41.9% [32]. When regarding risk factors, we showed a higher primary metronidazole resistance in female patients compared with male ones, most likely due to the wide use of imidazoles for gynaecological infection. In agreement with data presented by Glupczynski *et al *and Batnavala *et al*, metronidazole resistant isolates were found significantly more frequently in females than in males and in non-European natives than in subjects born in European countries [33,34]. The same cause was explained by Koletzko *et al *in their study where they found that metronidazole was widely used in Africa and Asia to treat parasitic diseases and gynaecological infections [35].

For children, the prevalence of clarithromycin primary resistance (18.8%) was in agreement with a French one where resistance to clarithromycin was detected in 18% [36], and slightly lower than those founded in a European prospective multicentre study and in Spain study where the rates were evaluated respectively to 20% and 21.1% [35,37], but more lower than that documented in an Argentinean study (29.1%) [38]. Our prevalence was lower than that reported by one previous Tunisian study conducted with 46 treated children, where the susceptibility testing was performed in 10 cases and where the rates of resistance to clarithromycin and metronidazole were respectively 30% and 90% [39].

When regarding the point mutations conferring resistance to clarithromycin, we found that A2143G was dominant followed by A2142G mutation, the A2142C was not detected. Domingo *et al *[40] and Pina *et al *[9] showed that the A2143G was more prevalent. De Francesco *et al *[41] reported that the A2143G was prevalent in their study and concluded that this mutation reduces markedly the eradication therapy efficacy. As for metronidazole resistance, our prevalence (25%) was much higher than that reported in 105 untreated children in Bulgaria (16.2%) [30], but was slightly higher than that documented from children in Argentina (23.9%), in Spain (23%) [37] and in a European study (23%) [35].

Overall, the resistance rate for clarithromycin was lower in adults than in children (14.6% vs. 18.8%), which points to an acquisition of the resistance during childhood. Indeed, macrolides are very often used nowadays to treat respiratory infections in young children. By contrast, for metronidazole the resistance rate was markedly higher in adults than in children (56.8% vs. 25%). Our results were not in agreement with those founded in a European study [35], where the rates were very similar in adults and children and where they concluded that the similar rate was in favour of transmission of a resistant strain to the children mostly from their mothers who widely used metronidazole to treat gynaecologic infections. In contrast to clarithromycin and metronidazole, no amoxicillin resistance was observed; this finding was similar to those determined by Kim *et al *[21] and Wolle *et al *[18] in their studies. This is despite the wide use of this antibiotic, both alone and combined with clavulanic acid for the treatment of respiratory tract infections or *H. pylori *infection in children and adults. However, resistance to amoxicillin has appeared in some countries with low frequencies and could increase. The determination of resistant strains to antibiotics by the E-test method has proven clinically reliable except in the case of metronidazole for which the E-test has a tendency to overestimate the presence of resistance. The reason is not known, but it may be that a pre-incubation of the media in an anaerobic atmosphere has been shown a factor that increasing metronidazole activity [42]. For that reason and for the lack of clinicobacteriological correlation, the Maastricht III 2005 Consensus Report discourages routine metronidazole susceptibility testing [43]. Then, further standardization should be performed regarding the medium, the inoculum and the growth atmosphere.

In conclusion, we believe that the widespread use of macrolides in our country for the treatment of upper respiratory tract infections could have an undesirable outcome in the emergence of clarithromycin resistant strains of *H. pylori*, reducing the success of therapy rate. Moreover, the rate of annual consumption of antibiotics in Tunisia was evaluated to 4% which is a risk factor for increasing resistant strains to antibiotics; when regarding macrolides, for example, the rate of consumption in Tunisia exceed 4 millions units per year [44]. The clarithromycin resistance decreases the effectiveness of antibiotic therapy and is the main risk factor for treatment failure: resistance reduces the efficacy of the first line therapy by up to 70% [24]. Among the Maastricht III consensus report [43], treatment should achieve an eradication rate of ≥ 80%. The threshold of clarithromycin resistance at which this antibiotic should not be used, or a clarithromycin susceptibility test should be performed, is 15-20% [43,45]. In Tunisia, physicians can continue the prescription of clarithromycin as a treatment for *H. pylori *infection in adults, in whom 14.6% of strains are resistant to this antibiotic, but in our paediatric population where the rate of this resistance was 18.8%, clarithromycin should not be used. Our study is the first to show that A2143G is the main genetic mutation involved in *H. pylori *clarithromycin resistance *in vivo*, thus suggesting that new therapeutic strategies are needed. When about metronidazole, we think that physicians might well refrain from using this antibiotic in order to prevent the emergence of more resistant strains especially because of the high rate of metronidazole resistant strains in Tunisia. In one study performed in North India, Bhatia *et al *[32] have concluded that imidazole-based eradication regimens should be abandoned in North India regardless of in vitro susceptibility results (41.9%).

Because of the emergence of resistant strains in our country, there is a clear need for a periodic evaluation of antibiotic consumption, particularly of clarithromycin in public hospitals and in private clinical sector.

There are no competing interests to declare for this study.

## Competing interests

There are no competing interests to declare for this study.

All enrolled patients were consent to take part in our research.

This prospective multicentre study was performed in Microbiology laboratory-Rabta University Hospital, Tunis, Tunisia in research unit UR04SP08.

## Authors' contributions

BMK was responsible for the collection of biopsies, *H. pylori *conventional culture, had carried out the molecular genetic studies, performed the statistical analysis, drafted the manuscript and participated in the design of the study. ZM and MA were participated in the design of the study. KS, KL, CS, MS, FM, ZS, LM, BJ, CI, BHM, SN, AA, FA, BMN, NT, MA and SMT were helped to the constitution of *H. pylori *strains collection, BC had helped to molecular genetic studies. Finally, FC had conceived of the study, participated in its design and coordination and helped to draft the manuscript. All authors have read and approved the final manuscript.
